# Novel highly divergent sapoviruses detected by metagenomics analysis in straw-colored fruit bats in Cameroon

**DOI:** 10.1038/emi.2017.20

**Published:** 2017-05-24

**Authors:** Claude Kwe Yinda, Nádia Conceição-Neto, Mark Zeller, Elisabeth Heylen, Piet Maes, Stephen Mbigha Ghogomu, Marc Van Ranst, Jelle Matthijnssens

**Affiliations:** 1 KU Leuven, Department of Microbiology and Immunology, Rega Institute for Medical Research, Laboratory of Viral Metagenomics, Leuven, Flemish Brabant 3000, Belgium; 2 KU Leuven, Department of Microbiology and Immunology, Rega Institute for Medical Research, Laboratory for Clinical and Epidemiological Virology, Leuven, Flemish Brabant 3000, Belgium; 3 University of Buea, Department of Biochemistry and Molecular Biology, Biotechnology Unit, Molecular and Cell Biology Laboratory, Buea, South West Region 237, Cameroon

**Keywords:** bat, feces, interspecies transmission, new genogroups, sapovirus

## Abstract

Sapoviruses (SaVs) belong to the *Sapovirus* genus, in the family *Caliciviridae*. They have been associated with gastroenteritis in humans and in pigs but not in other animals. In addition, some strains from pigs, chimpanzees and rodents show close sequence identity with human SaVs thereby suggesting the possibility of interspecies transmissions. Bats are known to be a major reservoir of zoonotic viruses, however, very little is known about the genetic diversity of SaVs in bats. To explore the genetic diversity of bat SaVs, fecal samples of *Eidolon helvum* and *Epomophorus gambianus* were treated according to the NetoVIR protocol and sequenced by Illumina technology. Nearly complete genome sequences of six highly divergent SaVs and one partial SaV (only VP1 region) were identified in *Eidolon helvum* and based on sequence identities and phylogenetic analysis, they potentially represent two novel genogroups, only distantly related to known SaVs. Furthermore, comparing these sequences with currently used screening primers and probes indicated that the novel SaVs would not be detected in routine epidemiological screening studies in humans in case an interspecies transmission would occur. Therefore, we designed and validated new primers that can detect both human and bat SaVs. In this study, we identified multiple novel bat SaVs, however, further epidemiological studies in humans are needed to unravel their potential role in gastroenteritis.

## INTRODUCTION

Sapovirus (SaV) particles were first detected in human diarrheic stool samples in 1976 in the United Kingdom using electron microscopy.^1^ Shortly thereafter they were recognized as a new gastroenteritis pathogen as reviewed by Oka and colleagues.^2^ Initially these viruses were known as 'typical human caliciviruses' or 'Sapporo-like' viruses. Since 2002, the International Committee on the Taxonomy of Viruses (ICTV) has assigned these viruses to the *Sapporo virus* species, *Sapovirus* genus, in the family *Caliciviridae*,^3^ together with four other genera: *Norovirus, Lagovirus, Vesivirus* and *Nebovirus* (www.ictvonline.org/). SaV particles are made up of a non-enveloped icosahedral capsid of 30–38 nm in diameter, with typical cup-shaped depressions on the surface.^4^ Inside the particle resides the positive-sense, single-stranded RNA genome of ~7.1–7.7 kb with a 3’- poly (A) tail. Generally, SaV genome contains two open reading frames (ORFs): ORF1 encodes for a large polyprotein containing the nonstructural proteins (helicase, VPg, protease, RNA-dependent-RNA-polymerase (RdRp)) followed by the major capsid protein, VP1; ORF2 is predicted to encode for the minor structural protein VP2.^5^ However, a third ORF has been found in some human^6, 7, 8, 9, 10, 11^ and bat^12^ SaV strains, although a known function is yet to be assigned. Until recently, SaVs were classified into five genogroups (GI–GV).^13^ With the exception of SaV GIII, SaV genogroups were further subdivided in one or multiple genotypes based on the full-length nucleotide (nt) sequence of VP1.^2, 13^ GI, GII, GIV and GV SaVs are known to infect humans, while GIII infects pigs.^10^ Recently, more SaVs have been detected in different animal species, and novel genogroups GVI–GXV were proposed on the bases that strains with ≥57% pairwise aa identity of VP1 should be in the same genogroup.^14^ A number of SaVs from animal hosts, such as chimpanzee^15^, pig^16, 17, 18^ and rodents^19^ have been found to cluster closely with human SaVs in GI, GV and GII, respectively. This clearly indicates the possibility of cross-species transmission of SaVs;^20^ however, this does not indicate the direction of the interspecies transmission. To further elucidate the zoonotic potential of SaVs, more human and animal samples need to be screened. Bats (order *Chiroptera*) constitute the second largest group of mammals after the order *Rodentia*^21^ and have recently been implicated as a reservoir of many different viruses including some highly pathogenic to humans.^22^ Little is known about the genetic diversity of SaVs in bats and currently bat SaVs have been described only in two studies conducted in China^12^ and Hungary.^23^ The bat SaVs found in these studies were highly divergent from other SaVs known thus far.

Here, we describe the nearly complete sequences of six highly divergent SaVs and one partial SaV obtained from the fecal samples of straw-colored fruit bats *(Eidolon helvum*) in Cameroon.

## MATERIALS AND METHODS

### Ethical authorization

Ethical authorization for the protocol and the use of animal samples was obtained from the Cameroon National Ethics Committee, Yaoundé. All animal experiments were performed in accordance with the Ministry’s National Ethics Committee guidelines. Administrative authorization (R.11/MINSANTE/SWR/RDPH/PS/290/157) was obtained from the Delegation of Public Health for South West Region, Cameroon.

### Sample collection

Fecal samples were collected from straw-colored fruit bats (*Eidolon helvum*) between December 2013 and May 2014 by a previously described method developed by Donaldson *et al.*^24^ Briefly, bats were captured in three different regions (Lysoka, Muyuka and Limbe) of the South West Region of Cameroon using mist nets around fruit trees and around human dwellings. Captured bats were retrieved from the net traps and held in paper sacks for 10–15 min, allowing enough time for the production of fresh feces. Sterile disposable spatulas were used to retrieve feces from the paper sacks, and placed into tubes containing 1 mL of universal transport medium (COPAN Diagnostics, Brescia, Italy). Labeled samples were put in ice and then transferred to the Molecular and Cell Biology Laboratory, Biotechnology Unit, University of Buea, Cameroon and stored at −20 °C. Later on, they were shipped to the Laboratory of Clinical and Epidemiological Virology, Leuven, Belgium and stored at −80 °C. Each captured bat was assessed to determine species, weight (g), forearm length (mm), sex, reproductive state, and age (adult or juvenile). Parameters such as body temperature, activity, photophobia were not measured. All captured bats were then marked by hair clipping to facilitate identification of recaptures. Trained zoologists used morphological characteristics to determine the species of the bats before they were released. None of the captured animals showed physical signs of disease.

### Sample preparation

Briefly, 25 pools (of two to five samples each) were made from 87 collected samples (85 from *Eidolon helvum* and two from *Epomophorus gambianus)* based on age, sex and location, and the pools were treated to enrich for viral particles using the NetoVIR protocol.^25^ Fecal suspensions (10% w/v in universal transport medium) were homogenized for 1 min at 3000 rpm with a MINILYS homogenizer (Bertin Technologies, Montigny-le-Bretonneux, France) and filtered using a 0.8 μm PES filter (Sartorius, Goettingen, Germany). The filtrate was then treated with a cocktail of Benzonase (Millipore, Billerica, MA, USA) (Novagen, Madison, WI, USA) and Micrococcal Nuclease (New England Biolabs, Ipswich, MA, USA) at 37 °C for 2 h to digest free-floating nucleic acids. Samples were extracted using the QIAamp Viral RNA Mini Kit (Qiagen, Hilden, Germany) according to the manufacturer’s instructions but without addition of carrier RNA to the lysis buffer. First and second strand synthesis and random PCR amplification for 17 cycles were performed using a slightly modified Whole Transcriptome Amplification (WTA2) Kit procedure (Sigma-Aldrich, St Louis, MO, USA). WTA2 products were purified with MSB Spin PCRapace spin columns (Stratec Biomedical, Birkenfeld, Germany) and the libraries were prepared for Illumina sequencing using a slightly modified version of the Nextera XT Library Preparation Kit (Illumina, San Diego, CA, USA). Sequencing of the samples was performed on a HiSeq 2500 platform (Illumina) for 300 cycles (2 × 150 bp paired ends).

### Genomic and phylogenetic analysis

Raw reads were trimmed for quality and adapters using Trimmomatic^26^ and were *de novo* assembled into contigs using SPAdes.^27^ Contigs were then annotated using DIAMOND with the sensitive option^28^ using the non-redundant (nr) database of GenBank. ORFs were identified with ORF Finder analysis tools and the conserved motifs in the amino acid (aa) sequences were identified with HMMER^29^ and NCBI's conserved domain database (CDD).^30^ According to the International Calicivirus Conference Committee, a complete VP1 region sequence is required to designate new genogroups or genotypes, and strains in the same genogroups have ≥57% pairwise aa identity of VP1.^13^ Based on these complete gene sequence (at least 1602 nt), our novel sequences and 51 other complete reference gene sequences were aligned in MEGA6^31^ using muscle and a phylogenetic tree generated using maximum-likelihood method, based on the LG+G+I+F substitution model (after testing for the best DNA/protein model in MEGA6). Sequences used in the phylogenetic analysis were representatives of the different genogroups and/or genotypes, complemented with all known bat SaVs, and SaVs which had been previously reported as potentially involved in zoonotic events.^14, 19, 32, 33^ Amino acid sequence pairwise distances were also calculated in MEGA6 using transition and transversion substitution models. Sequences from the novel SaVs were submitted to GenBank with the following accession numbers: KX759618–KX759624.

### Primer design and validation

Degenerate screening primers (SaV4529F: CCN TCD GGV ATG CCW TTY AC and SaV5165R: TYR CCC TCC ATY WCA AAC AC) were designed based on an alignment of 26 human SaVs (representative members of all genogroups) and 12 bat SaVs. These primers were used to screen 12 SaV-positive samples (eight human samples and four bat samples) by PCR. The following PCR conditions were used: an initial reverse transcription step at 50 °C for 30 min was followed by a PCR activation step at 95 °C for 15 min, 39 cycles of amplification, and a final extension step for 10 min at 72 °C in a Biometra T3000 thermocycler (Biometra). The cycle conditions for denaturation, annealing and elongation were 94 °C for 45 s, 58 °C for 45 s and 72 °C for 1 min, respectively. PCR products were run in polyacrylamide gel, stained with EtBr and visualised under UV-light.

## RESULTS

**Figure 1:**
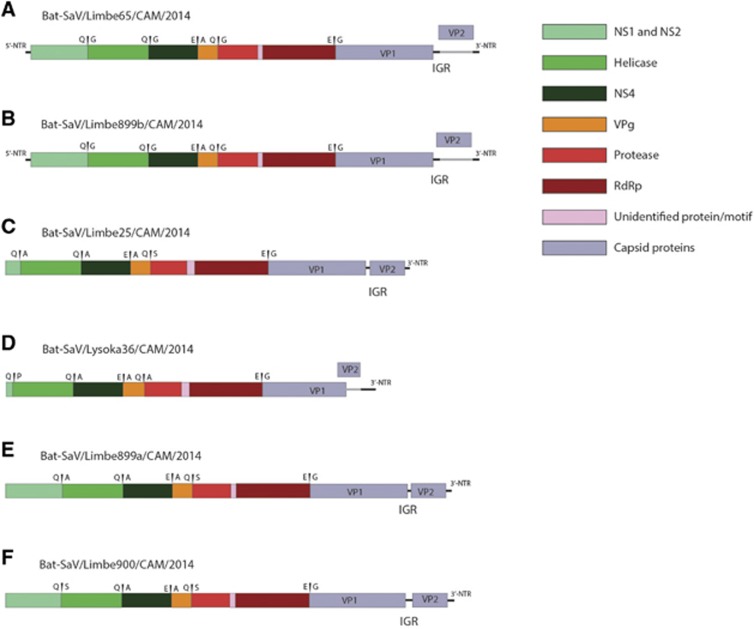
Genome organization of novel sapovirus strains from straw-colored fruit bats. (**A**, **B**) SaV strains Limbe65 and Limbe899b with non-overlapping ORF1 and ORF2 in different reading frames; (**D**) SaV strain Lysoka36 with an overlapping ORF1 and ORF2; (**C**, **E**, **F**) SaV strains Limbe25, Limbe899a and Limbe900 with non-overlapping ORF1 and ORF2 in the same reading frame.

Fecal samples from apparently healthy straw-colored fruit bats were pooled (three to five samples per pool) based on sex age and location and sequenced by Illumina next-generation sequencing (NGS) technology. Fourteen out of the twenty-five pools contained SaV reads, ranging from 0.1% to 6.3% of the non-phage viral reads. All SaV-positive pools were from the straw-colored fruit bat (*Eidolon helvum*) while the lone pool of Gambian epauletted fruit bat (*Epomophorus gambianus)* contain no SaV reads. These positive pools were from both sexes and age groups and were identified in samples from two out of three localities (Limbe and Lysoka) (Details in Table 1). We were able to obtain two full-length genomes sequences (Limbe65 and Limbe899b); two near-complete genomes (Limbe900 and Limbe899a) with at least the full-length coding region; and two incomplete genomes (Limbe25 and Lysoka36) without a complete coding region. Also, a partial sequence of the major capsid gene (VP1) of Limbe894 was obtained. Bat SaVs Limbe65 and Limbe899b have sequence lengths of 7475 and 7474 nt, with a G+C content of 48.8% and 46.3%, respectively. Their genomes contain two non-overlapping ORFs flanked by 5’-UTRs of 9 nt and 3’-UTRs (52 nt and 51 nt, respectively) (Figures 1A and 1B). Both have an ORF1 of 6819 nt, encoding the nonstructural proteins and the major capsid protein VP1. The ORF2 (492 nt) encodes for a minor structural protein VP2. The other four nearly complete genomes (Figures 1C–1F) show similar genomic characteristics except that Lysoka36 (Figure 1D), has overlapping ORF1 and ORF2 sequences (an overlapping region of 102 nt). In addition, the two ORFs of SaVs Limbe25, Limbe900 and Limbe889a are located in the same reading frame, whereas those of Limbe65, Limbe899a and Limbe899b can be found in different frames.

Table 2 shows the characteristic aa motifs conserved in all caliciviruses: 2C-like NTPase motif 
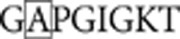
; VPg motifs KGKTK and 
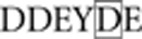
; 3C-like protease motif GDCG; 3D-like RdRP motifs WKGL, KDEL, DYSKWDST, GLPSG and YGDD; and VP1 motif PPG. Only minor deviations were observed in the novel SaVs: in the 2C-like NTPase motif (
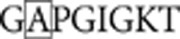
), the A is replaced with either I or P and in the VPg motif (
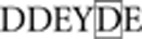
), the last D is replaced by an E in all the novel sequences.

On the basis of the complete *VP1* gene of all bat SaVs, human SaVs and animal SaVs closely related to human SaVs, the nt and aa similarity percentages of representative members of the different genogroups and/or genotypes were calculated (Supplementary Table S1). Hong Kong *Hipposideros Pomona* bat SaVs (TLC34, TLC39 and TLC58) identified by Tse *et al*^12^ shares 99%–100% aa identity among each other, are only 37%–42% similar to other SaVs, and were previously assigned to genotype XIV.^14^ On the other hand, two unpublished Chinese strains, GX2012 (from *Rhinolophus sinicus*) and JX2010 (from *Myotis myotis*), are only 50% similar to each other on the aa level while M63 (a Hungarian strain from *Myotis daubentonii*)^23^ shares 71% identity (67% nt identity) with JX2010. Therefore, the genotype numbers GXVI and GXVII were tentatively assigned to the strains GX2012, and JX2010 and M63, respectively. The sequences of the seven bat SaVs identified in Cameroon have an aa identity ranging from 31% to 41% (40%–49% nt identity) with all other SaVs and only 37%–41% (46%–48% nt identity) with other bat SaVs. On the basis of sequence similarity, the Cameroonian strains fall into two groups. The first group has two nearly identical pairs: Limbe65 and Limbe899b; Limbe 25 and Limbe 899a (99%–100% nt and aa identity) and between these pairs, there is an aa similarity of 77% (71% nt similarity). The rest of the strains (Limbe900, Lysoka36 and Limbe894) are more closely related to each other, with an aa similarity ranging from 61% to 78% (62%–72% nt similarity) among each other. These two groups share only 49%–54% aa (53%–57% nt) similarity among them. According to the classification scheme proposed by Oka *et al*,^14^ our bat SaVs represent two new genogroup within the *Sapovirus* genus. We tentatively placed them into genogroups GXVIII and GXVIX, respectively.

Phylogenetic analysis was performed using the aa sequences of the entire VP1 region of our sequences and 55 sequences representing genogroup and/or potential zoonotic strains. The human SaVs were found in four clusters, representing four genogroups (GI, GII, GIV and GV) while the porcine SaVs were found not only in genogroup GIII, but also in genogroups GV-GXI.

**Figure 2:**
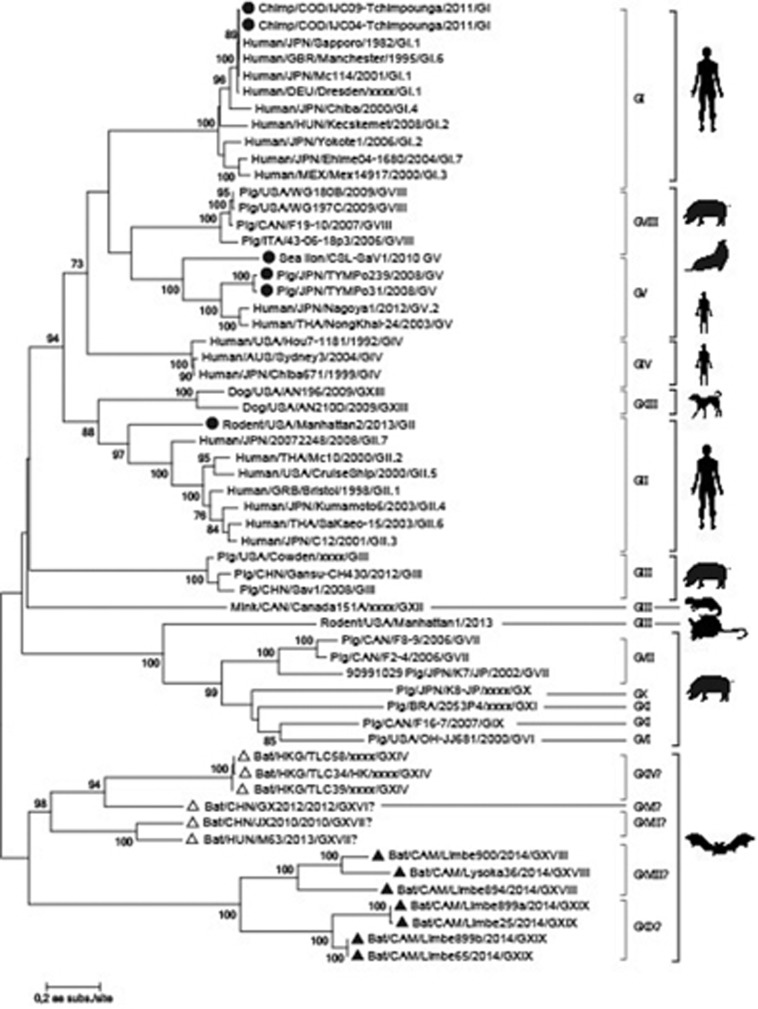
Maximum-likelihood phylogenetic tree, based on a VP1 amino acid sequence alignment of Limbe65, Limbe899a, Limbe899b, Limbe25, Limbe900, Limbe894 and Lysoka36, and 55 other representative SaVs strains. Previously known bat SaVs are indicated with open triangles, whereas those described in this paper are indicated with filled triangle. Filled circles are strains with the potential history of cross between species. The numbers at the internal nodes represent the bootstrap probabilities (in percent), as determined for 1000 iterations. Only bootstrap values greater than 70% are shown. The scale bar indicates the genetic distance (amino acid substitutions per site).

The phylogenetic analysis revealed a single monophyletic clade of bat SaVs, containing two major clusters (Figure 2). The first cluster contains SaV strains from Asia and Hungary divided over one established and two putative genogroups. Based on aa similarity, bat strains from Hong Kong (TLC39, TLC58 and TLC34) constitute genogroup GXIV. SaV strain GX2012 (a distantly related Chinese strain), is the lone strain in the putative genogroup XVI, while JX2010 (another Chinese strain) together with Hungarian strain M63 are found in the putative genogroups XVII. The African SaVs cluster contained the SaVs strains described in this paper. This cluster is further subdivided into two putative novel genogroups (Limbe65, Limbe899a, Limbe899b and Limbe25 on one hand and Limbe900, Limbe894 and Lysoka36 on the other hand). The phylogenetic tree based on the *RdRP* gene (Supplementary Figure S1) confirmed the observations based on the *VP1* gene. Comparing these Cameroonian bat sequences to partial RdRP from African wildlife SaVs^34^ indicated only 46%–57% nt indentity, however, they were not included in the phylogenetic tree because only very short sequences (<300 nt) of the RdRPs were available in GenBank.

**Figure 3:**
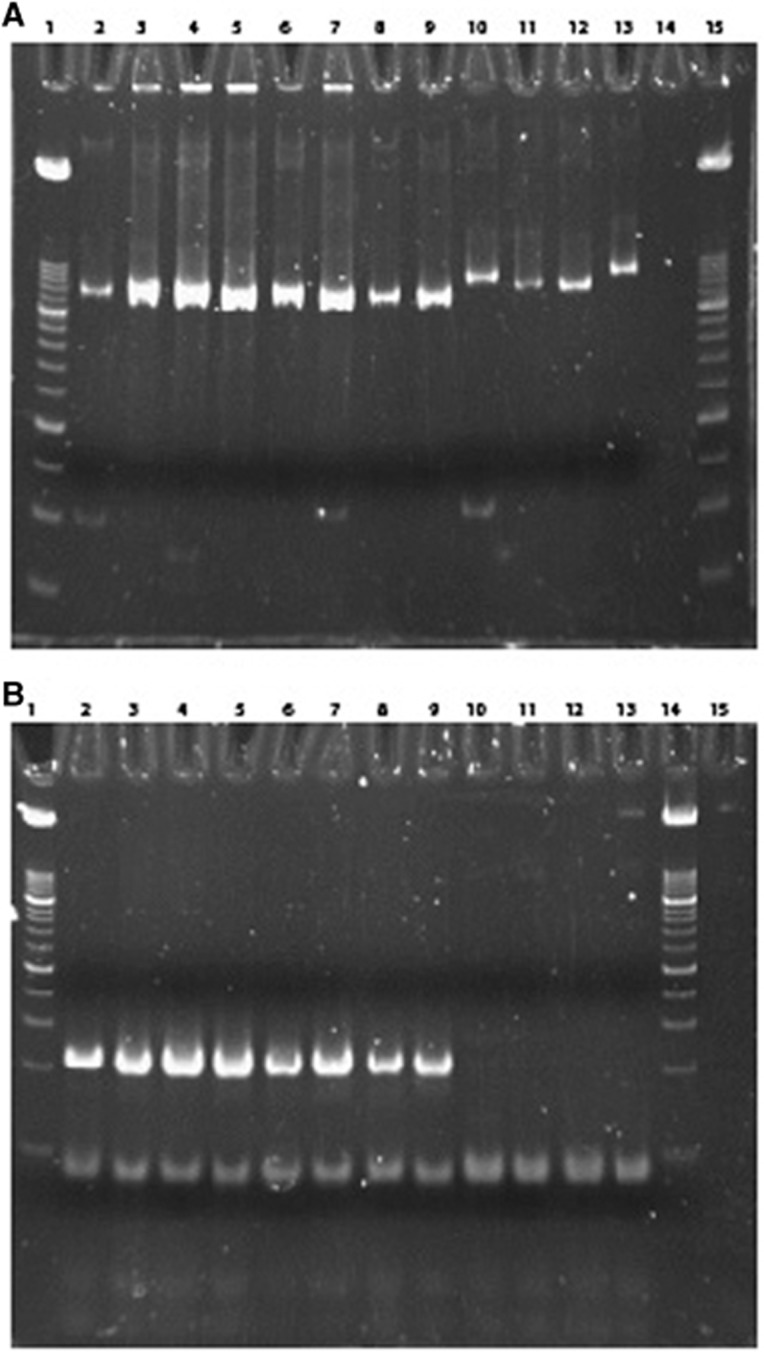
Polyacrylamide gel electrophoresis of the PCR product amplified with novel primers (**A**) and currently used primers (**B**). Wells 1 and 14 or 15: DNA Molecular Weight Marker VIII; wells 2–9: human samples positive for SaV (genogroups GI.1, GI.3, GII.3, GII.5, GII.6, GIV and GV; wells 10–13: bat samples positive for SaV (novel putative genogroups GXVIII and GXIX).

Given the high genetic divergence of the bat SaVs described here and human SaVs, it is doubtful that the currently used human screening primers will also detect these newly found SaVs in case of interspecies transmission from bats to humans. To answer this question, we compared the frequently used screening primers (SaV124F, SaV1F, SaV5F and SaV1245R) and probes (SaV124TP and SaV5TP)^35^ with sequences of the novel bat strains (Table 3). It showed that the percentage similarity ranged from 25% to 66.7% for forward primers and 94.7% to 100% for the reverse primers. Considering the probes, the percentage similarity was only 42.9% and 57.1%. These primers and probes are unlikely to pick up SaVs from bats, and therefore we designed novel screening primers based on the RdRP segment of human and bat sequences, to screen both human and bat fecal samples positive for SaVs. The used samples contain human SaVs of genogroup GI.1, GI.3, GII.3, GII.5, GII.6, GIV and GV and the novel putative bat SaVs of genogroups GXVIII and GXIX. As expected, the novel primers detected both human and bat SaVs while the currently used primers detected only human SaVs (Figure 3).

## DISCUSSION

SaV are known human gastroenteritis etiological agents and the identification of animal SaV strains closely related to human SaVs has raised awareness for the possibility of SaV interspecies transmissions. Due to an increasing interest in bats as a reservoir of zoonotic viruses and the fact that bat SaVs have only been sporadically reported, there is an eminent need to fully explore bat species for this virus and its zoonotic potential. Here, we collected and metagenomically screened 87 bat samples from fruit bats in Cameroon. SaV reads were present in samples from both juvenile and adult, as well as male and female bats, suggesting that viral presence does not seem to be restricted by either age or sex. We were able to obtain six nearly complete genomes and one capsid protein (VP1) of SaVs in Cameroonian bats, each of which encoded two ORFs. SaVs of genogroup GI, GIV, and GV have been reported to encode a third ORF overlapping the *VP1* gene,^10, 36^ which was also detected in a previous study in bats, although their ORF3 did not overlap with the VP1 of ORF1.^12^ The fact that the third ORF was not identified in our novel strains and other animal SaVs^18^ points to the fact that the protein encoded by this ORF in bats and human GV strains might not be mandatory. To ascertain the function of the protein, cell culture propagation, functional and knockout studies are required. Furthermore, the GC content of the newly described bat SaVs falls within the typical GC range of the genus *Sapovirus* (49.0% to 53.6%). However, SaVs identified previously in Pomona roundleaf bat (*Hipposideros pomona)* showed a relatively high GC content (up to 60.2%).^12^ In addition, the overlap of ORF1 and ORF2 in the genome of Lysoka36 (Figure 1D), to the best of our knowledge is novel. This highlights the existence of a high genomic diversity of bat SaVs, although the true diversity is most likely much larger. Further studies are needed to better understand the SaV diversity in bats and other animals.

SaVs have been associated with gastroenteritis in people of all ages,^37, 38^ and in pigs,^39^ but not in other animals. It is not clear whether these viruses cause disease in bats, since the adult and young bats from which they were identified appeared healthy based on physical examination (for wounds or abnormalities on the body or wings) and no signs of white-nose syndrome were observed. This is not surprising as most bat viruses known today were discovered in apparently healthy bats, suggesting that they may have specific immune system or antiviral activity against virus infections,^40^ making them effective reservoirs of viruses.

These novel African SaVs clearly formed two phylogenetic clusters: the first cluster is made of Limbe65, Limbe899a, Limbe899b and Limbe25 and the second is made of Limbe900, Limbe894 and Lysoka36. Each cluster differs from the other and all other SaV genogroups by at least 40% aa identity and therefore constitute two new genogroups within the SaV genus, tentatively assigned as genogroups GXVIII and GXVIX, respectively. SaV genogroups GI, GII, GIV and GV infect humans while all other SaV genogroups (GIII and GVI–GXVII) have been identified in animals. Phylogenetically, some of the strains isolated from animals are related to human SaVs. Two SaVs (IJC09 and IJC04) isolated from chimpanzees in The Republic of Congo clustered closely with human G1 SaVs. Given that these chimps were living in close contact to humans (in the sanctuary) the authors suggested that there might have been a recent cross-species transmission of these viruses.^15^ Also three porcine SaV strains (TYMPo239, TYMPo031 and WG194D-1) and one California sea lion SaV were identified independently and are relatively closely related to human SaV GV.^14, 32, 33^ Lastly, based on the pairwise nt distances between the rodent strain Manhattan/Ro-SaV2 and the closest human SaVs (GII.1 to GII.7), it can be concluded that this rodent strain also belongs to the genogroup GII SaV. This suggests that these human and rat strains have a common origin, most likely reflecting an interspecies transmission event in a (distant) past.^19^ All these data from animal studies suggest that interspecies transmission of these viruses might have occurred in the past and as a consequence, raise the possibility of circulating animal SaVs strains in humans. Given that the bats we sampled live in closely proximity to humans in the region, there might have been an ample opportunity for such interspecies transmission to occur between bats and humans. However, most of the epidemiological SaVs studies described so far used conserved screening primers in PCR assay to identify and sequence the VP1 or VP1-RdRp region. Due to the nt divergence of bat SaVs observed here, even if there were zoonotic strains from bats circulating in humans, they might not be detected by the existing primers. Therefore, the novel primers presented here will be able to detect both zoonotic strains especially from bats. Further, to fully delineate the zoonotic capacity of these bat SaVs, *in vitro* studies in cell cultures would be very useful, as well as screening human samples around the same region for bat SaVs.

## Figures and Tables

**Table 1:** Number of SaV reads and percentages of total number of non-phage viral reads

Pool	Sex	Age	Location	# non-phage viral reads	# of sapovirus reads	% SaV reads	Other viruses in the pool
P1^a^	M	Adult	Lysoka	7068	250	3.5	2, 9 and 12
P2	M	Adult	Lysoka	71955	625	0.9	1, 9 and 11
P3	F	Adult	Lysoka	81322	75	0.1	9 and 14
P4	F	Adult	Lysoka	72953	0	0.0	4, 6, 7, 9, 12 and 15
P5	M	Adult	Moyuka	1206	0	0.0	8, 9, and 16
P6	F	Adult	Moyuka	450	0	0.0	9
P7	M	Adult	Limbe	7156	0	0.0	4, 9, 11, 14 and 15
P8	M	Adult	Limbe	52278	570	1.1	11, 14 and 15
P9	F	Adult	Limbe	20866	224	1.1	2 and 4
P10	F	Adult	Limbe	46295	56	0.1	2, 4, 6, 11 and 15
P11^a^	M	Young	Limbe	60856	3827	6.3	4, 6 and 15
P12	M	Young	Limbe	787	0	0.0	2, 4, 6, 11 and 15
P13	M	Young	Limbe	2016	0	0.0	4, 6 and 15
P14^a^	F	Young	Limbe	50567	452	0.9	2, 3, 12 and 17
P14^b^	F	Young	Limbe	188953	14068	7.4	2, 3, 12 and 17
P15	F	Young	Limbe	108240	42	0.0	3, 12, 15 and 17
P16^c^	F	Adult	Lysoka	110542	0	0.0	3 and 9
P17^a^	F	Adult	Lysoka	217016	471	0.2	9, 11 and 14
P18	F	Adult	Lysoka	1370	0	0.0	9, 11, 14, 16 and 17
P19	F	Adult	Moyuka	2569	0	0.0	9, 11, 12, 14 and 17
P20	F	Adult	Limbe	609	0	0.0	4, 6, 11, 14, 15, and 17
P21	F	Adult	Limbe	2923	0	0.0	4, 6, 11, 14, 15 and 17
P22^d^	F	Young	Limbe	568732	7295	1.3	2 and 17
P23^a^	M	Adult	Limbe	620965	2563	0.4	4
P24	M	Young	Limbe	1853	84	4.5	1, 2, 3, 5, 11, 14, 15 and 17
P25	F	Adult	Limbe	15355	0	0.0	3, 4, 8, 11, 14, 15 and 17

Abbreviations: next-generation sequencing, NGS; sapovirus, SaV.

a Pool from which SaV reads were described.

b Pool on which NGS was repeated after pretreatment of the extracted viral nucleic acids with DNsase to obtain a relative enrichment of RNA virus (sapovirus) reads.

c Pool of samples from *Epomophorus gambianus*, no SaV reads. Other viruses are presented with numbers from 1 to 17 as follows: 1=*Astroviridae*, 2=*Caliciviridae*, 3=*Circoviridae*, 4=*Coronaviridae*, 5=*Hepeviridae*, 6=*Herpesviridae*, 7=*Nodaviridae*, 8=*Papillomaviridae*, 9=*Partitiviridae*, 10=*Paramyxoviridae*, 11=*Parvoviridae*, 12=*Picobirnaviridae*, 14=*Reoviridae*, 15=*Retroviridae*, 16=*Totiviridae*, 17=*Tymovirales*.

d Pool with two sapovirus strains.

**Table 2:**
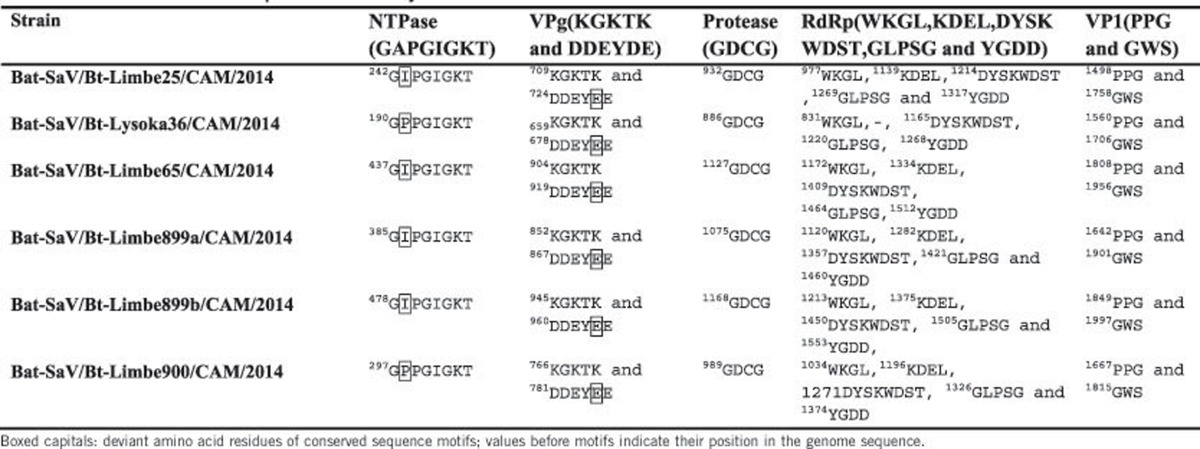
Motifs of functional proteins of newly described bat SaVs

**Table 3:**
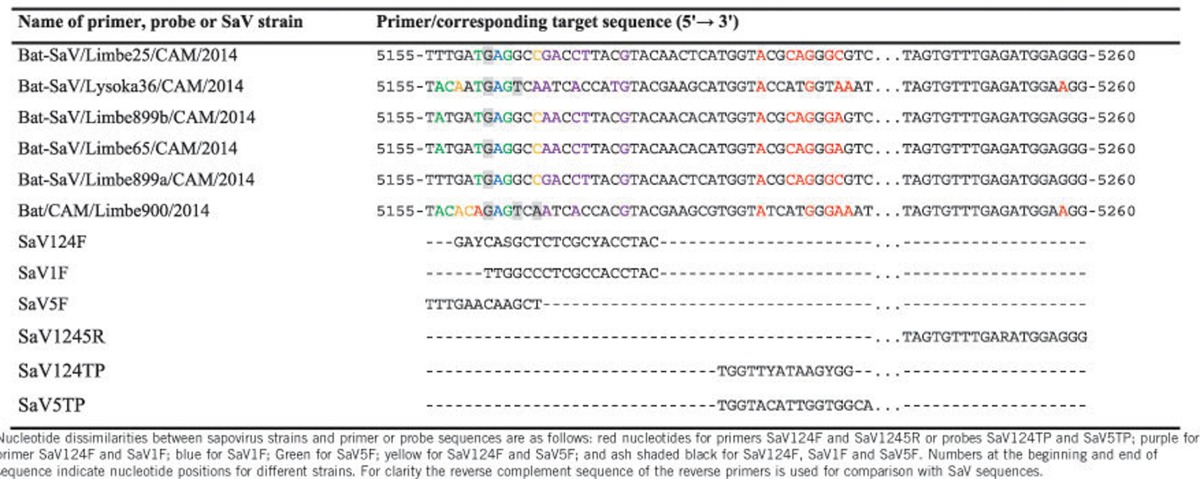
Nucleotide comparison between the primers sequences used for human sapovirus screening with the corresponding region of the newly identified bat sapovirus strains
