# Supplementary material for: Novel highly divergent sapoviruses detected by metagenomics analysis in straw-colored fruit bats in Cameroon
Source: Emerg Microbes Infect. 2017 May 24;6(5):e38–. doi: 10.1038/emi.2017.20 (PMC5520483; doi:10.1038/emi.2017.20)

**Supplementary Figure S1** Maximum Likelihood phylogenetic tree based on an RdRP amino acid sequence alignment of Limbe65, Limbe899a, Limbe899b, Limbe25, Limbe900, Limbe894 and Lysoka36, and 43 other SaVs strains. Previously known bat SaVs are indicated with open triangles, whereas those described in this paper are indicated with filled triangles. Filled circles are strains with the potential to cross between species. The numbers at the internal nodes represent the bootstrap probabilities (in percent), as determined for 1000 iterations. Only bootstrap values greater than 70% are shown. The scale bar indicates the genetic distance (amino acid substitutions per site).


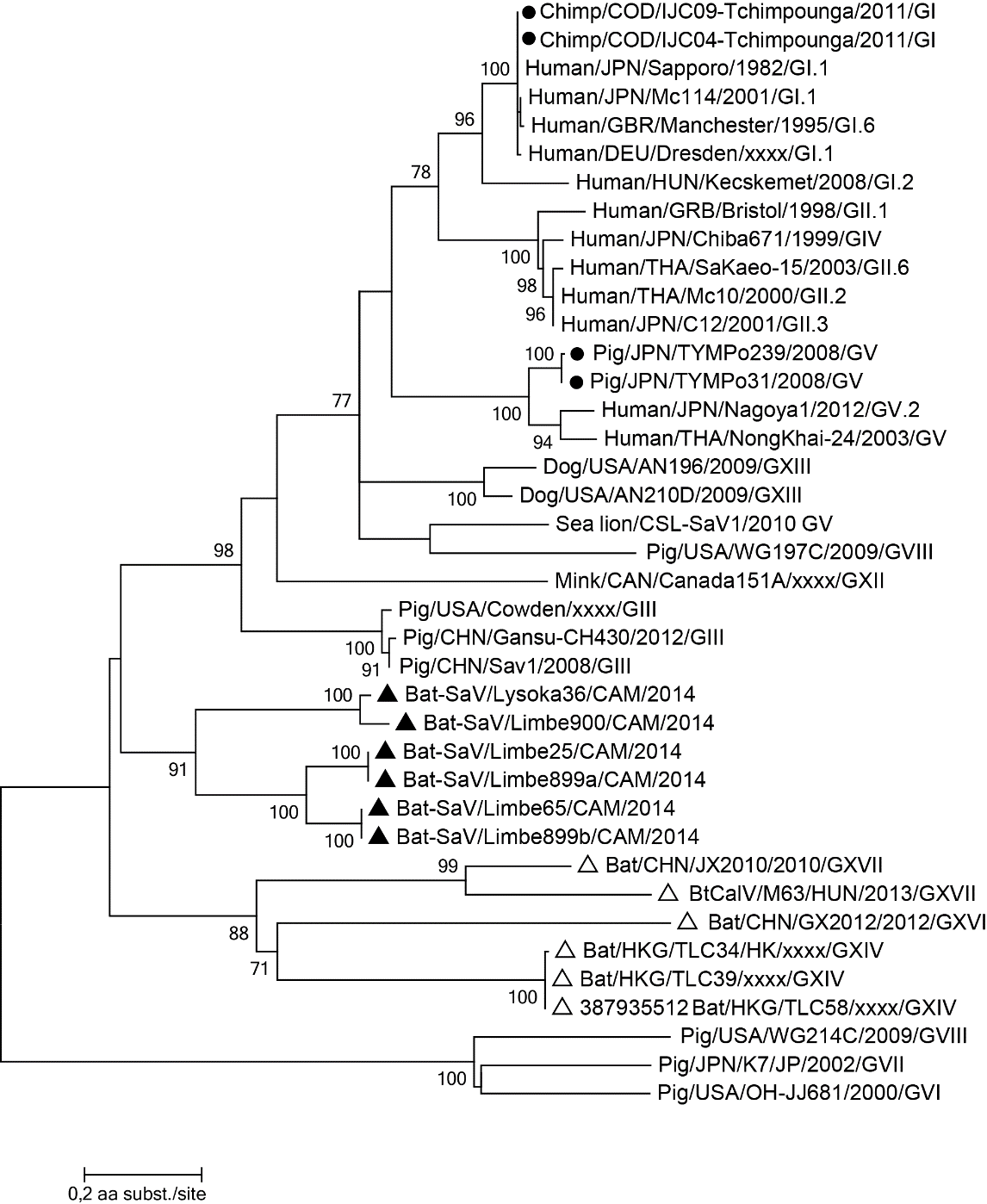

Supplement: Supplementary Figure S1 [file emi201720x1.docx]
