# Supplementary material for: Novel highly divergent sapoviruses detected by metagenomics analysis in straw-colored fruit bats in Cameroon
Source: Emerg Microbes Infect. 2017 May 24;6(5):e38–. doi: 10.1038/emi.2017.20 (PMC5520483; doi:10.1038/emi.2017.20)
Supplement: Supplementary Table S1 [file emi201720x2.docx]

# Supplementary Table S1 Nucleotide (upper right) and amino acid (lower left) percentage similarity of VP1 gene of Cameroonian SaVs, other bats SaVs, representative members of different genogroup and/or genotypes, and those SaVs reported to have zoonotic potential. Black lines demarcate genogroups based on the cutoff of 57% amino acid similarity.
